# HDAC6 Restricts Influenza A Virus by Deacetylation of the RNA Polymerase PA Subunit

**DOI:** 10.1128/JVI.01896-18

**Published:** 2019-02-05

**Authors:** Huan Chen, Yingjuan Qian, Xin Chen, Zhiyang Ruan, Yuetian Ye, Hongjun Chen, Lorne A. Babiuk, Yong-Sam Jung, Jianjun Dai

**Affiliations:** a MOE Joint International Research Laboratory of Animal Health and Food Safety, College of Veterinary Medicine, Nanjing Agricultural University, Nanjing, Jiangsu Province, China; b Key Laboratory of Animal Bacteriology, Ministry of Agriculture, College of Veterinary Medicine, Nanjing Agricultural University, Nanjing, Jiangsu Province, China; c Shanghai Veterinary Research Institute, Chinese Academy of Agricultural Sciences, Shanghai, China; d University of Alberta, Edmonton, Alberta, Canada; University of Southern California

**Keywords:** deacetylation, HDAC6, Influenza A virus, PA, RNA polymerase activity

## Abstract

The life cycle of influenza A virus (IAV) is modulated by various cellular host factors. Although previous studies indicated that IAV infection is controlled by HDAC6, the deacetylase involved in the regulation of PA remained unknown. Here, we demonstrate that HDAC6 acts as a negative regulator of IAV infection by destabilizing PA. HDAC6 binds to and deacetylates PA, thereby promoting the proteasomal degradation of PA. Based on mass spectrometric analysis, Lys(664) of PA can be deacetylated by HDAC6, and the residue is crucial for PA protein stability. The deacetylase activity of HDAC6 is required for anti-IAV activity, because IAV infection was enhanced due to elevated IAV RNA polymerase activity upon HDAC6 depletion and an HDAC6 deacetylase dead mutant (HDAC6-DM; H216A, H611A). Finally, we also demonstrate that overexpression of HDAC6 suppresses IAV RNA polymerase activity, but HDAC6-DM does not. Taken together, our findings provide initial evidence that HDAC6 plays a negative role in IAV RNA polymerase activity by deacetylating PA and thus restricts IAV RNA transcription and replication.

**IMPORTANCE** Influenza A virus (IAV) continues to threaten global public health due to drug resistance and the emergence of frequently mutated strains. Thus, it is critical to find new strategies to control IAV infection. Here, we discover one host protein, HDAC6, that can inhibit viral RNA polymerase activity by deacetylating PA and thus suppresses virus RNA replication and transcription. Previously, it was reported that IAV can utilize the HDAC6-dependent aggresome formation mechanism to promote virus uncoating, but HDAC6-mediated deacetylation of α-tubulin inhibits viral protein trafficking at late stages of the virus life cycle. These findings together will contribute to a better understanding of the role of HDAC6 in regulating IAV infection. Understanding the molecular mechanisms of HDAC6 at various periods of viral infection may illuminate novel strategies for developing antiviral drugs.

## Abstract (precis)

Influenza A virus (IAV) continues to threaten global public health due to drug resistance and the emergence of frequently mutated strains. Thus, it is critical to find new strategies to control IAV infection. Here, we discover one host protein, HDAC6, that can inhibit viral RNA polymerase activity by deacetylating PA and thus suppresses virus RNA replication and transcription. Previously, it was reported that IAV can utilize the HDAC6-dependent aggresome formation mechanism to promote virus uncoating, but HDAC6-mediated deacetylation of α-tubulin inhibits viral protein trafficking at late stages of the virus life cycle. These findings together will contribute to a better understanding of the role of HDAC6 in regulating IAV infection. Understanding the molecular mechanisms of HDAC6 at various periods of viral infection may illuminate novel strategies for developing antiviral drugs.

## INTRODUCTION

Influenza A virus (IAV) continually poses severe threats to human and animal health and to economic development due to seasonal epidemics and occasional pandemics. Because of its rapid antigenic alterations and adaptation to new hosts, prevention and treatment are difficult. IAV has 8 negative-sense RNA segments: two surface glycoproteins (hemagglutinin [HA] and neuraminidase [NA]), two matrix proteins (M1 and M2), a nonstructural protein (NS1), a nuclear export protein (NS2), a nucleoprotein (NP), and an RNA polymerase complex heterotrimer including PA (polymerase acidic protein), PB1 (polymerase basic protein 1), and PB2 (polymerase basic protein 2) (1). During infection, virus enters the cell through endocytosis and fusion, and then, the viral ribonucleoproteins (vRNPs) are released into the cytoplasm and transported into the nucleus to start transcription and replication. IAV RNA polymerase regulates virus transcription and replication. During the transcription process, capped and polyadenylated mRNA is synthesized by RNA polymerase using 5′-capped RNA primers (2, 3). During the replication process, a cRNA replication intermediate is produced and serves as a template for new vRNA synthesis.

PA is a polymerase acidic protein that plays an essential role in the formation of the heterotrimer complex (4). The N-terminal domain of PA possesses endonuclease activity, and the C-terminal domain is required for PB1 association. Studies have shown that PA protein can be modified by phosphorylation (5) and ubiquitination (6). However, the effects of posttranslational modifications of PA protein on RNA polymerase activity are not well known.

Histone deacetylases (HDACs) are a family of enzymes that function in gene expression, chromatin remodeling, protein stability, and transport (7, 8). Histone deacetylase 6 (HDAC6) is a unique deacetylase enzyme in the HDAC family. It possesses two deacetylase domains and a zinc finger motif (ubiquitin binding), mostly localizes in the cytoplasm, and mediates various cellular processes by deacetylating nonhistone substrates, including heat shock protein 90 (HSP90), α-tubulin, and cortactin (9–11). HDAC6 also interacts with polyubiquitinated or misfolded proteins, leading to aggresome formation and protein degradation (12). Deacetylase domain 1 (DD1) of HDAC6 has been shown to possess ubiquitin E3 ligase activity (13). Moreover, HDAC6 is implicated in virus infection. For example, HIV-1 fusion and infection are inhibited by HDAC6-mediated deacetylation of α-tubulin (14). IAV can utilize the aggresome-processing machinery mediated by HDAC6 to facilitate its uncoating and infection (15). However, trafficking of IAV components is limited by deacetylation of microtubules via HDAC6 (16). The direct function of HDAC6 on viral proteins remains unknown.

In this study, we showed that HDAC6-mediated PA deacetylation reduces its protein stability. We also demonstrated that Lys(664) of PA is targeted for acetylation, as well as ubiquitination. In addition, we found that HDAC6-mediated deacetylation of the PA Lys(664) residue is required for its degradation. Importantly, we showed that IAV RNA transcription and replication are suppressed or enhanced in the presence or absence of HDAC6, respectively, along with altered IAV RNA polymerase activities, which rely on the catalytic activity of HDAC6. Therefore, our findings provide previously unidentified insights into the molecular mechanisms responsible for HDAC6 acting as an anti-IAV host factor that can suppress IAV genome transcription and replication through deacetylation of PA to inhibit the IAV RNA polymerase activity.

## RESULTS

### HDAC6 is associated with PA.

**FIG 1:**
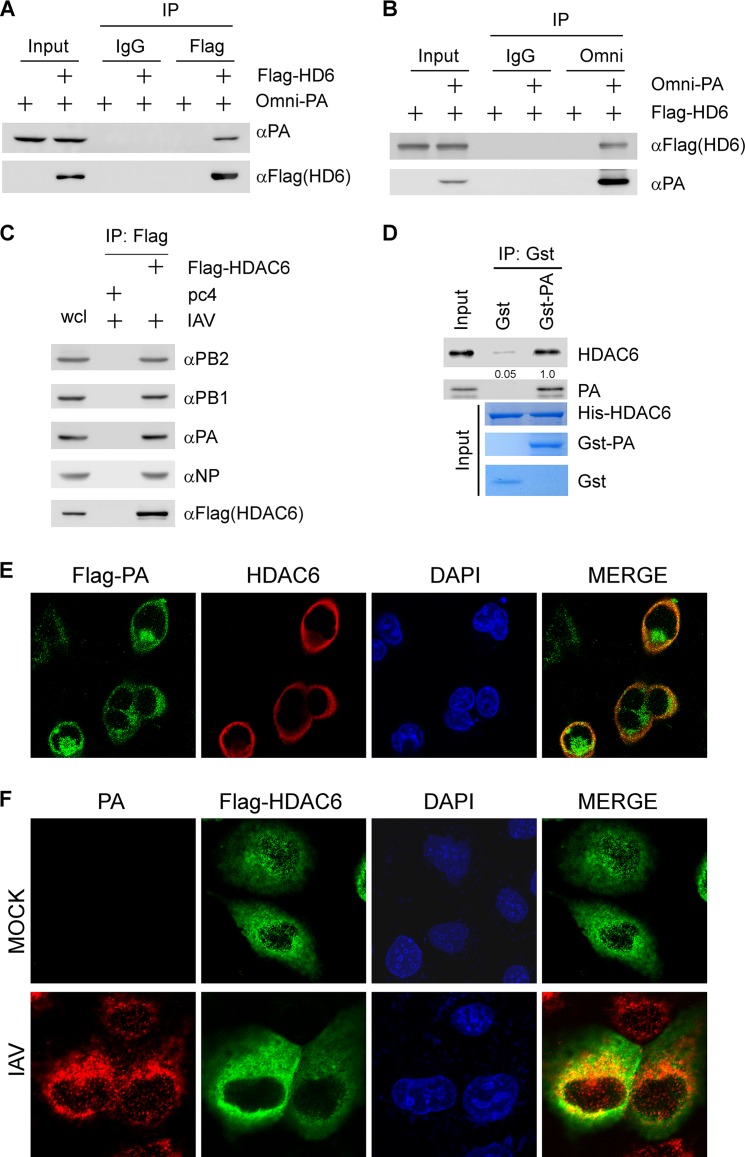
HDAC6 interacts with PA, one subunit of the IAV RNA polymerase complex. (A) 293T cells were cotransfected with Flag-HDAC6 or empty vector and Omni-PA, and whole cell lysates were immunoprecipitated (IP) with Flag antibody or control IgG. The immunocomplexes were then used to detect Omni-PA and Flag-HDAC6 by immunoblotting with the indicated antibodies. (B) The experiment was performed as for panel A, except that whole-cell lysates were immunoprecipitated with Omni antibody or control IgG. (C) MDCK cells were transfected with Flag-HDAC6 or control vector for 24 h, followed by infection with WSN (multiplicity of infection [MOI] = 1.0) for 24 h, and then immunoprecipitated with Flag antibody. The immunocomplexes were then used to detect Flag-HDAC6 and RNA polymerase complex by immunoblotting with the indicated antibodies. (D) His-HDAC6, GST-PA, and GST proteins were purified from E. coli. His-HDAC6 was incubated with GST or GST-PA immobilized on glutathione-Sepharose beads for 4 h. The bound protein was eluted with SDS sample buffer and analyzed by immunoblotting. (E) 293T cells were cotransfected with Flag-PA and HDAC6. After 36 h, the cells were fixed and stained with Flag antibody and Alexa 488-conjugated goat anti-mouse IgG antibody (green) and then stained with HDAC6 and Alexa 555-conjugated goat anti-rabbit IgG antibody (red). The nuclei were stained with DAPI (blue). The images were acquired with a Nikon confocal microscope. (F) MDCK cells were transfected with Flag-HDAC6 for 36 h, followed by infection with WSN (MOI = 2.0) for 7 h, and then fixed and stained with Flag antibody and Alexa 488-conjugated goat anti-mouse IgG antibody (green), followed by staining with PA antibody and Alexa 555-conjugated goat anti-rabbit IgG antibody (red). The nuclei were stained with DAPI (blue).

HDAC6 is involved in IAV uncoating via mediating aggresome formation and indirectly inhibiting trafficking of viral components by regulating the acetylation states of α-tubulin (15, 16). Additionally, HDAC6 was known to be associated with viral NP. This finding led us to investigate whether HDAC6 regulates IAV RNA polymerase activity. To test this, we first examined whether HDAC6 could bind to the IAV (strain WSN) RNA polymerase complex. Flag-HDAC6 and Omni-PA were cotransfected into 293T cells, which were then immunoprecipitated with anti-Flag and anti-Omni, respectively. We showed that overexpressed Omni-PA was present in the anti-Flag (HDAC6) immunocomplex (Fig. 1A). Conversely, ectopically expressed Flag-HDAC6 was detected in the anti-Omni (PA) immunocomplex (Fig. 1B). These findings led us to test whether HDAC6 was associated with other IAV RNA polymerase components, such as PB1 and PB2. We found that upon immunoprecipitation with anti-Flag (HDAC6), viral proteins PB1 and PB2 were detected in HDAC6 immunocomplexes (see Fig. S1A and B in the supplemental material). Interaction between HDAC6 and NP was also detected in 293T cells (see Fig. S1C). To detect the interaction between HDAC6 and IAV RNA polymerase components upon virus infection, MDCK (Madin-Darby canine kidney epithelial) cells were infected with WSN for 24 h. The immunoprecipitation result showed that HDAC6 can interact with IAV RNA polymerase components upon virus infection (Fig. 1C). To demonstrate the direct interaction between HDAC6 and PA, a glutathione *S*-transferase (GST) pulldown experiment was performed with recombinant GST-PA and His-HDAC6. We found that the GST-PA protein directly interacted with recombinant His-HDAC6 (Fig. 1D). Next, we examined the subcellular localization of HDAC6 and PA under a confocal microscope. Flag-PA and HDAC6 were cotransfected into 293T cells, which were then immunostained by anti-Flag fluorescein isothiocyanate (FITC) and anti-HDAC6 tetramethyl rhodamine isocyanate (TRITC) antibodies, respectively. Immunofluorescence confocal microscopy revealed that ectopically expressed Flag-PA was colocalized with HDAC6 (Fig. 1E). To further examine the subcellular localization of HDAC6 and PA, MDCK cells were transfected with Flag-HDAC6 and then infected with IAV (WSN). The data showed that HDAC6 can be colocalized with WSN IAV PA protein (Fig. 1F).

### The deacetylase activity of HDAC6 is required for PA degradation.

**FIG 2:**
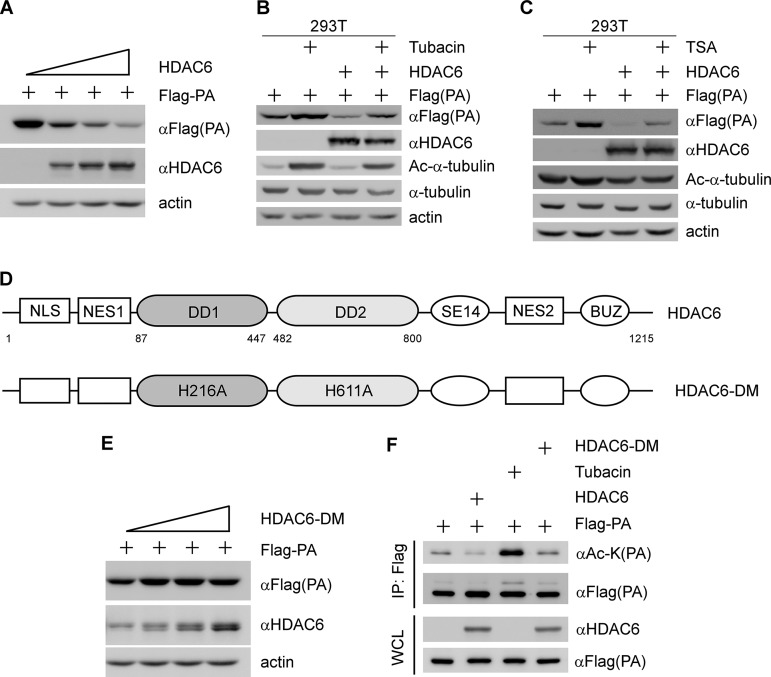
The deacetylase activity of HDAC6 is required for PA degradation. (A) 293T cells were cotransfected with a fixed amount (500 ng) of Flag-PA and increasing amounts of HDAC6 for 36 h, and then the cell lysates were analyzed by immunoblotting with the indicated antibodies. (B) 293T cells were transfected with Flag-PA, along with or without HDAC6, for 20 h and then left untreated or treated with tubacin (10 μM) for 17 h. Flag-PA, HDAC6, Ac-α-tubulin, α-tubulin, and actin were detected by immunoblotting. (C) The experiment was performed as for panel B, except that TSA was used. (D) Schematic diagram of HDAC6 and HDAC6-DM. (E) The experiment was performed as for panel A, except that HDAC6-DM was used. (F) an *in vitro* deacetylation assay was performed. 293T cells were transfected with Flag-PA, HDAC6, or HDAC6-DM separately for 36 h, and then Flag-PA cell lysates were treated with tubacin (10 μM) or coincubated with HDAC6 (or HDAC6-DM) cell lysates. The cell lysates were immunoprecipitated with Flag antibody and then analyzed by immunoblotting with the indicated antibodies.

Previously, it was reported that N-terminal acetylated proteins can be stabilized by avoiding the proteasome-dependent degradation pathway (17). Indeed, HDAC6 is a deacetylase enzyme and can bind to the WSN IAV RNA polymerase complex. To investigate whether HDAC6 can affect RNA polymerase complex protein degradation, Flag-PA was transiently expressed in 293T cells along with an increased dose of HDAC6. We showed that the level of exogenous PA protein was gradually decreased by HDAC6 overexpression in a dose-dependent manner (Fig. 2A). The degradation of PA can be recovered by the proteasome inhibitor MG132 (see Fig. S2A in the supplemental material). However, HDAC6 was not able to downregulate the levels of PB1, PB2, and NP (see Fig. S2B to D). To further confirm the HDAC6-mediated PA degradation, 293T cells were cotransfected with PA or PA together with HDAC6 and then treated with the HDAC6-specific inhibitor tubacin or the pan-HDAC inhibitor trichostatin A (TSA). We found that PA protein can be downregulated by HDAC6 overexpression and upregulated by tubacin treatment (Fig. 2B). A similar result was observed after TSA was used to treat 293T cells (Fig. 2C). To further examine whether the deacetylase activity of HDAC6 is critical for PA degradation, an HDAC6 deacetylase dead mutant (HDAC6-DM; H216A, H611A) was constructed (Fig. 2D) (12). The results showed that HDAC6-DM was not able to downregulate the exogenous level of PA (Fig. 2E). These findings led us to test whether HDAC6 can promote the deacetylation of PA by transfecting 293T cells with Flag-PA, along with HDAC6 or HDAC6-DM, and then immunoprecipitating the Flag-PA proteins with anti-Flag. We showed that acetylated PA was dramatically decreased with HDAC6, but not with HDAC6-DM (Fig. 2F). Conversely, acetylated PA was recovered by tubacin treatment (Fig. 2F). These results suggest that HDAC6 deacetylates PA protein, depending on its deacetylase activity.

### Identification of lysine residues in PA for deacetylation by HDAC6.

**FIG 3:**
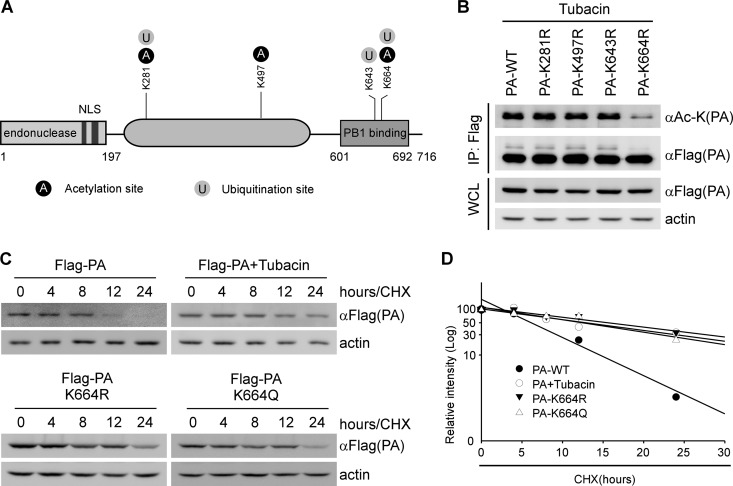
HDAC6 mediates the deacetylation of PA at Lys(664). (A) Schematic diagram of PA modification. NLS, nuclear localization signal. (B) 293T cells were transfected with Flag-PA or its acetylation dead mutants as indicated and then treated with tubacin (10 μM) for 17 h. Flag antibody was used to immunoprecipitate the wild type or acetylation dead mutants of Flag-PA, which were then analyzed by immunoblotting with the indicated antibodies. WCL, whole-cell lysates. (C) 293T cells were transfected with the indicated plasmids, followed by tubacin or DMSO treatment for 17 h. The cells were then treated with CHX (10 μg/ml) at the indicated time points. PA and actin were detected by immunoblotting with the indicated antibodies. (D) Calculated relative half-lives of PA, PA-K664R, and PA-K664Q, using the data from panel C. The percent intensity (log_10_) was plotted versus time.

Next, mass spectrometry (MS) was performed to determine whether or which Lys residues on the PA are required for deacetylation. 293T cells were transfected with Flag-PA and HDAC6 separately, and then, Flag-PA cell lysates were treated with tubacin or coincubated with HDAC6 cell lysates. The cell lysates were immunoprecipitated with Flag antibody and then Coomassie stained. The Coomassie staining gel for mass spectrometry is shown in Fig. S3A in the supplemental material. We found that several Lys residues of PA could be acetylated and ubiquitinated. The modification sites of PA are shown in Fig. 3A. Among the potentially modified residues, Lys(664) of PA could be acetylated by tubacin treatment and deacetylated by HDAC6 (see Fig. S3B). Interestingly, the mass spectrometry result showed that Lys(664) of PA could be modified by acetylation (see Fig. S3B) and ubiquitination (see Fig. S3C). Based on the results, we generated PA mutants that carried one substitution with Arg at Lys(281), Lys(497), Lys(643), and Lys(664), along with a Flag tag. These PA mutants were then transfected in 293T cells, along with tubacin treatment. Three PA mutants that carried Arg substitutions (K281R, K497R, and K643R) were found to still be acetylated (Fig. 3B). In contrast, the level of acetylation of one PA mutant (K664R, referred to here as PA K664R) was dramatically decreased (Fig. 3B). These results suggest that HDAC6 deacetylates PA protein at Lys(664).

Because PA protein is one of the IAV RNA polymerase subunits, its stability is important for maintaining RNA polymerase activity. This led us to investigate whether the acetylation status of PA can affect its stability. To address this, Flag-PA (PA-WT [wild type]), an acetylation mimic PA mutant (PA K664Q), and an acetylation dead PA mutant (PA K664R) were expressed in 293T cells with or without tubacin treatment. We showed that the relative half-life of PA protein was markedly increased by tubacin treatment or acetylation mimic and dead mutations (Fig. 3C). Similar results were obtained when the relative level of PA protein was quantified and normalized with actin (Fig. 3D). These results are consistent with the idea that HDAC6-mediated PA deacetylation promotes its proteasomal degradation.

### HDAC6 suppresses IAV transcription and replication.

**FIG 4:**
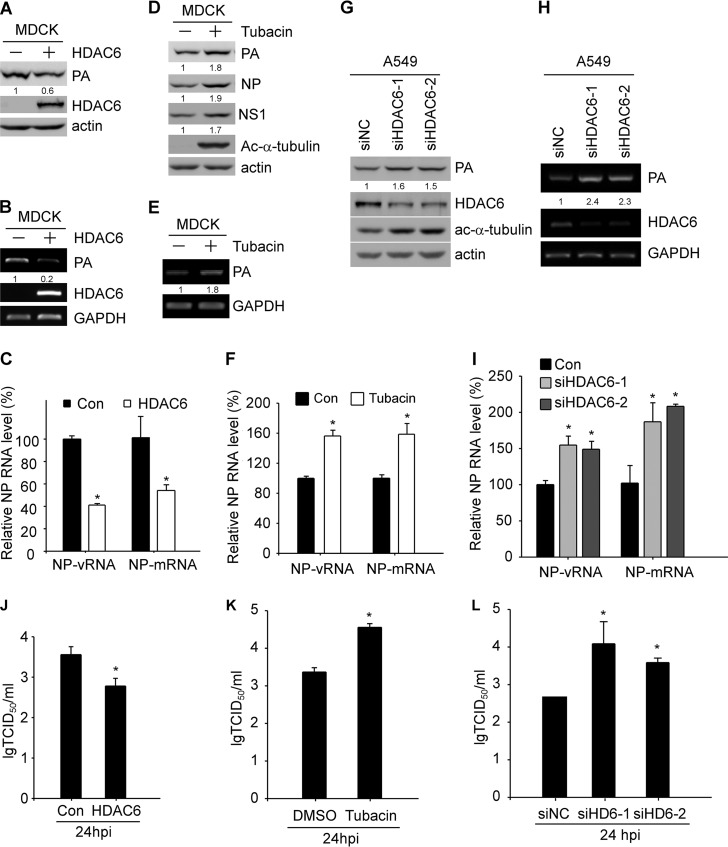
HDAC6 regulates IAV RNA transcription and replication. (A) MDCK cells were transfected with HDAC6 or empty vector for 36 h, followed by WSN infection (MOI = 2) for 3 h. PA, HDAC6, and actin were detected by immunoblotting. (B) The experiment was performed as for panel A except that the samples were collected for RNA purification. PA, HDAC6, and GAPDH mRNAs were detected by RT-PCR. (C) The experiment was performed as for panel B, and NP mRNA and vRNA levels were quantified by Q-RT-PCR. Con, control. (D) MDCK cells were pretreated with tubacin (1 μM) for 2 h and subsequently infected with WSN (MOI = 2) for 3 h. The cell lysates were prepared for immunoblotting to detect PA, NP, Ac-α-tubulin, and actin. (E) The levels of PA and GAPDH were detected by RT-PCR using RNA purified from samples as for panel D. (F) The experiment was performed as for panel E, except that cells were collected for RNA extraction. The levels of transcripts for PA, HDAC6, and GAPDH were measured by RT-PCR. (G) A549 cells were transfected with nontargeting scrambled siRNA or targeting HDAC6 siRNA for 48 h, followed by virus infection (WSN; MOI = 2) for 3 h. The viral proteins PA, HDAC6, Ac-α-tubulin, and actin were detected by immunoblotting. (H) The experiment was performed as for panel E, and NP mRNA and vRNA levels were quantified by Q-RT-PCR. (I) The experiment was performed as for panel H, except that the levels of NP mRNA and vRNA were quantified by Q-RT-PCR. (J) MDCK cells were transfected with HDAC6 or empty vector for 24 h, followed by WSN infection (MOI = 0.1) for 24 h. The supernatants were titrated in MDCK cells. (K) MDCK cells were pretreated with tubacin (1 μM) for 2 h, followed by WSN infection (MOI = 0.1) for 24 h. The supernatants were titrated in MDCK cells. (L) A549 cells were transfected with nontargeting scrambled siRNA or targeting HDAC6 siRNA for 48 h, followed by virus infection (MOI = 0.1) for 24 h. The supernatants were titrated in MDCK cells. Student's *t* test was used for statistical analysis. A *P* value of less than 0.05 was considered statistically significant (*, *P < *0.05). The error bars indicate standard deviation from three independent experiments.

During IAV infection, the virus can utilize many host factors to facilitate its replication. This has been shown by HDAC6 being required for efficient uncoating (15), HSP90 being involved in the assembly and transportation of RNA polymerase subunits (18), and CRM1 promoting the nuclear export of the viral ribonucleoprotein complex (19). In late stages of virus infection, HDAC6 plays an opposite role in virus release by inhibiting viral components trafficking to the cell membrane (16). The acetylation of the HIV Tat protein is critical for its transcriptional activity (20). In this study, we showed that PA can be deacetylated and downregulated by HDAC6 as described above. Thus, we examined whether HDAC6 could regulate IAV transcription and replication. Because HDAC6 is involved in uncoating and secretion at different stages of IAV infection (15, 16), we reconfirmed the data showing that the secretion levels of IAV were increased by tubacin treatment at 24 h postinfection (hpi) (see Fig. S4A and B in the supplemental material), consistent with a previous report (16). Therefore, we performed our experiment at 3 hpi, before the stage of virus secretion. MDCK and A549 cells were transfected with HDAC6 and then infected with WSN IAV. We showed that viral proteins were decreased upon HDAC6 overexpression in both MDCK (Fig. 4A) and A549 (see Fig. S5A in the supplemental material) cells. We also found that the levels of viral PA mRNA and NP mRNA/vRNA were dramatically decreased by HDAC6 overexpression (Fig. 4B and C). The decreased viral protein and RNA were also observed at different infection time points (see Fig. S5B, C, and D). To confirm the effect of HDAC6 on viral replication, MDCK and A549 cells were infected with IAV with or without the HDAC6-specific inhibitor tubacin. As expected, viral proteins were increased in MDCK and A549 cells upon tubacin treatment (Fig. 4D; see Fig. S6A and B in the supplemental material). Next, the viral PA mRNA and NP mRNA/vRNA levels were measured by reverse transcription (RT)-PCR and quantitative real-time PCR (Q-RT-PCR), respectively. We found that the levels of mRNA and vRNA were both increased in IAV-infected cells upon tubacin treatment (Fig. 4E and F). The increased viral mRNA and vRNA were also observed at 12 hpi (see Fig. S6C). To further confirm our results, we analyzed the effect of HDAC6 knockdown on IAV replication. A549 cells were transfected with HDAC6 small interfering RNA (siRNA) (siHDAC6), and we showed that PA protein was increased in A549 cells upon HDAC6 knockdown (Fig. 4G). Next, the viral PA mRNA level and NP mRNA/vRNA were measured by RT-PCR and Q-RT-PCR, respectively. The result also showed that the levels of mRNA and vRNA were remarkably upregulated by HDAC6 knockdown (Fig. 4H and I). Finally, viral titers were also determined upon overexpression or depletion of HDAC6 or tubacin treatment. We found that viral titers were decreased by HDAC6 overexpression (Fig. 4J) and increased when inhibition of HDAC6 deacetylase activity or knockdown of HDAC6 occurred (Fig. 4K and L). These data suggested that HDAC6 plays a negative role in IAV transcription and replication.

### Deacetylase activity of HDAC6 is required for IAV suppression.

**FIG 5:**
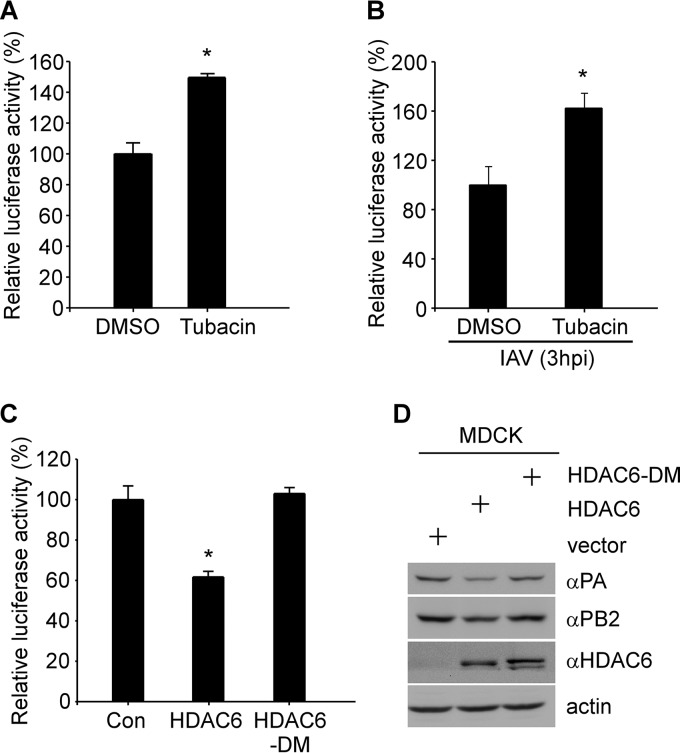
HDAC6 deacetylase activity is critical to inhibit IAV infection. (A) Luciferase activity was measured in the presence of the WSN IAV RNA polymerase complexes, along with DMSO or tubacin treatment, as described in Materials and Methods. (B) Luciferase activity was measured following IAV infection, along with DMSO or tubacin treatment. (C) Overexpression of HDAC6 or HDAC6-DM, along with WSN IAV RNA polymerase complexes; the relative luciferase signals are shown. Student's *t* test was used for statistical analysis. A *P* value of less than 0.05 was considered statistically significant (*, *P < *0.05). (D) MDCK cells were transfected with empty vector, HDAC6, or HDAC6-DM for 36 h, followed by WSN infection (MOI = 2) for 3 h. The error bars indicate standard deviation from three independent experiments.

The IAV RNA polymerase complex is formed by protein-protein interactions among individual polymerase subunits (i.e., PA, PB1, and PB2). The PA subunit is involved in the assembly of the RNA polymerase complexes (4) and is essential for transcriptional and replicative processes (21). Based on our findings, it is likely that HDAC6 affects the IAV RNA polymerase activity. To address this possibility, the WSN IAV minigenome reporter, in which the firefly luciferase gene in the negative sense is flanked by the cRNA promoter of the IAV NP segment, was cloned between a human RNA polymerase I promoter and the hepatitis D virus ribozyme (22). The luciferase experiment was performed using the influenza virus minigenome assay (22, 23). MDCK cells were cotransfected with PA, PB1, PB2, NP, pPolI-NP-firefly, and pCMV-RL, followed by tubacin treatment. We found that IAV RNA polymerase activity was significantly increased upon tubacin treatment (Fig. 5A). To validate the effect of tubacin on the IAV RNA polymerase activity, MDCK cells were transfected with pPolI-NP-firefly and then pretreated with tubacin. The dual-luciferase assay was performed by IAV infection at 3 hpi. Similarly, the IAV RNA polymerase activity was also increased upon tubacin treatment (Fig. 5B). The IAV RNA polymerase activity could be suppressed by HDAC6 overexpression, whereas overexpression of HDAC6-DM failed to downregulate IAV RNA polymerase activity (Fig. 5C). We also demonstrated that HDAC6 leads to attenuation of IAV replication and, hence, its respective protein (i.e., PA and PB2) levels, whereas in HDAC6-DM-transfected MDCK cells, IAV replication was recovered (Fig. 5D).

**FIG 6:**
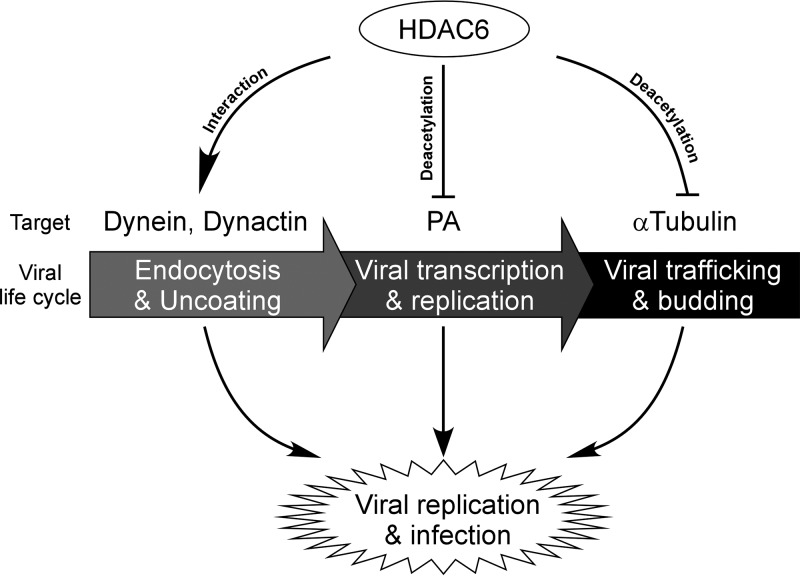
Model for the role of HDAC6 during IAV infection. HDAC6 interacts with dynein and dynactin to facilitate IAV uncoating. The IAV RNA polymerase activity is repressed by HDAC6-mediated PA deacetylation. HDAC6 inhibits IAV progeny release by suppressing the trafficking of viral HA protein.

Therefore, we can speculate that the deacetylase activity of HDAC6 is required for anti-IAV activity. Collectively, our results show that HDAC6 plays a role in inhibiting IAV replication by suppressing IAV RNA polymerase activity (Fig. 6).

## DISCUSSION

In this study, we identified HDAC6 as a key player in the molecular mechanism that governs the unique ability of the IAV RNA polymerase complex to rapidly produce large amounts of IAV progeny. HDAC6 is a deacetylase enzyme expressed in a wide range of cells and is involved in various physiological functions, including IAV infection (15, 16). In this study, we clarified a previously unidentified role of HDAC6 in the suppression of IAV replication. HDAC6 was found to physically interact with IAV PA protein, further deacetylating PA at Lys(664) to suppress IAV RNA polymerase activity and subsequently inhibit IAV replication, which depended on the deacetylase catalytic activity of HDAC6. Conversely, IAV RNA polymerase activity was enhanced by the HDAC6-specific inhibitor tubacin or HDAC6 depletion.

Posttranslational modifications (PTMs) play an important role in various biological processes. Ubiquitin regulates IAV entry and replication (24–26) and upregulates IAV polymerase function (27); phosphorylation of IAV NS1 impairs its interferon-antagonistic activity (28), and phosphorylation of M1 is crucial for its nuclear import (29); the acetylation of IAV HA is essential for the formation of fusion pores (30), and IAV infection leads to acetylation of α-tubulin to promote polarized protein transport (31). Recently, it was reported that N-terminal acetylation of PB1, PA, NP, M1, M2, NS1, and NEP was detected by mass spectrometry (32), but the functional effects of acetylation remain unknown. To our knowledge, this is the first demonstration that when PA is deacetylated by HDAC6, the ability of PA to assemble IAV RNA polymerase subunits is lost, resulting in the proteasomal degradation of PA. We also found that PA can be acetylated at Lys(281), Lys(497), and Lys(664). Among those acetylation sites, HDAC6 can promote deacetylation of PA at Lys(664). Interestingly, we demonstrated that Lys(664) of PA could also be ubiquitinated (Fig. 3B) and that HDAC6-mediated PA degradation occurs via the proteasomal degradation pathway (see Fig. S2A). Although we demonstrated that HDAC6 promotes PA destabilization, it remains possible that HDAC6 may ubiquitinate PA for degradation. Indeed, it was reported that the DD1 domain is responsible for ubiquitin E3 ligase activity (13). Therefore, future studies are warranted to determine whether HDAC6 and other ubiquitin E3 ligases catalyze PA ubiquitination.

According to previous reports, HDAC6 exhibits opposite characteristics in IAV infection and replication. HDAC6 can promote IAV entry by enhancing IAV uncoating at early stages (15). Meanwhile, HDAC6 also can be regulated by virus infection. At a late stage of IAV infection, HDAC6 can be cleaved by caspase 3 at its BUZ domain, which is important for HDAC6’s role in IAV-induced apoptosis (33). However, IAV trafficking and release can be inhibited by downregulating the transporting of viral components to the plasma membrane through acetylated microtubules (16). HDAC6 is a cytoplasmic enzyme that mediates various biological functions by its deacetylase and ubiquitin binding activities. HDAC6 is involved in immune synapse formation (34, 35), misfolded-protein degradation (12), stress response (36), and virus infection. Previous studies on the regulation of virus by HDAC6 have mainly focused on two aspects: (i) mediating viral transport and fusion and the release of viral components through modulating cytoskeletons and plasma membrane dynamics (14–16) and (ii) modulating the antiviral immune response by host cells (37, 38). Recently, it was reported that HDAC6 deacetylates Lys(909) of RIG-I, which results in activation of RIG-I to recognize viral RNA (39). In our study, we found that HDAC6 can suppress IAV RNA polymerase activity directly by downregulating PA protein.

In conclusion, our data demonstrate a new regulatory mechanism of the IAV RNA polymerase complex through the physical interaction between HDAC6 and PA. The identification and characterization of HDAC6-mediated deacetylation of PA suggest that deacetylase enzymes play a critical role during IAV infection. Further characterization of a stepwise assembly of the IAV RNA polymerase complex by HDAC6 will be valuable for the development of novel antiviral strategies.

## MATERIALS AND METHODS

### Cell culture and plasmids.

A549 (human lung adenocarcinoma; ATCC) cells, MDCK (ATCC) cells, and 293T (human embryonic kidney 293; ATCC) cells were cultured in Dulbecco’s modified Eagle medium (DMEM) (Invitrogen) supplemented with 8% fetal bovine serum (FBS) (Gibco), 1% penicillin and streptomycin (MDBio) at 37°C in a 5% CO_2_ incubator. cDNA encoding full-length human HDAC6 was subcloned in frame into the mammalian expression vector pcDNA4 (Invitrogen), with or without an N-terminal Flag tag, or pEGFP-C1. NP, PA, PB1, and PB2 of WSN IAV were subcloned into pcDNA3 (Invitrogen) with an N-terminal Flag or Omni tag (ASMTGGQQMGRDLYDDDDK). PA and HDAC6 were then subcloned into the prokaryotic expression vectors pGEX-4T-1 (Amersham Pharmacia Biotech) and pET-30a (Novagen). The pPolI-NP-firefly plasmid was generated as previously described (22).

Replacement of lysine(s) with arginine(s) or glutamine in PA was generated by site-directed mutagenesis. The following primers were used: PA-K281R-F (5′-GTT CTC AGC GGT CCA GAT TCC TGC TGA TGG AT-3′) and PA-K281R-R (5′-ATC CAT CAG CAG GAA TCT GGA CCG CTG AGA AC-3′), PA-K497R-F (5′-AGG AGG GAA GGC GAA GGA CCA ATT TGT ACG G-3′) and PA-K497R-R (5′-CCG TAC AAA TTG GTC CTT CGC CTT CCC TCC T-3′), PA-K643R-F (5′-AAC TTT ATT GGC AAG GTC GGT ATT CAA CAG C-3′) and PA-K643R-R (5′-GCT GTT GAA TAC CGA CCT TGC CAA TAA AGT T-3′), PA-K664R-F (5′-TCA GCT GAA TCA AGA AGA CTG CTT CTT ATC GTT C-3′) and PA-K664R-R (5′-GAA CGA TAA GAA GCA GTC TTC TTG ATT CAG CTG A-3′), and PA-K664Q-F (5′-TCA GCT GAA TCA AGA CAA CTG CTT CTT ATC GTT C-3′) and PA-K664Q-R (5′-GAA CGA TAA GAA GCA GTT GTC TTG ATT CAG CTG A-3′). An HDAC6 dead mutant was cloned as previously described by Yao et al. (Addgene plasmid no. 30483) (12). The following primers were used: HDAC6-H216A-F (5′-GGC CTC CTG GAC ATG CCG CCC AGC ACA GTC TTA-3′) and HDAC6-H216A-R (5′-TAA GAC TGT GCT GGG CGG CAT GTC CAG GAG GCC-3′) and HDAC6-H611A-F (5′-GTC CCC CAG GAC ACG CCG CAG AGC AGG ATG CAG-3′) and HDAC6-H611A-R (5′-CTG CAT CCT GCT CTG CGG CGT GTC CTG GGG GAC-3′). The HDAC6-H216/611A (HDAC6-DM) construct was generated by combining HDAC6-H216A and HDAC6-H611A mutants.

### Antibodies and reagents.

The antibodies used in this study included mouse anti-Flag and rabbit anti-actin antibodies purchased from Sigma; polyclonal rabbit anti-PA antibody obtained from GeneTex; and anti-NP, anti-NS1, anti-HDAC6, anti-acetyl (Ac)-α-tubulin, anti-α-tubulin, and anti-Omni antibodies purchased from Santa Cruz Biotechnology. The anti-acetyl lysine antibody was obtained from PTM Bio, and the NP-FITC antibody was purchased from Thermo Fisher. Goat anti-mouse IgG conjugated with horseradish peroxidase (HRP) and goat anti-rabbit IgG conjugated with HRP were purchased from Millipore. Tubacin, TSA, and cycloheximide (CHX) were purchased from Selleck. Lipofectamine 2000 and protease inhibitor cocktails (PIC) were purchased from Thermo Fisher Scientific.

### Virus infection and titer determination.

Influenza A virus [A/WSN/1933(H1N1) and A/PuertoRico/8/34(H1N1)] was cultured with MDCK cells as described above. The cells were infected with IAV and incubated at 37°C for 1 h. After incubation, the cells were washed with phosphate-buffered saline (PBS) and transferred to DMEM containing 1 μg/ml *N*-p-tosyl-l-phenylalanine chloromethyl ketone (TPCK)-treated trypsin (Worthington Biochemicals) and 0.3% bovine serum albumin (BSA) (Genview). The virus titer was determined by 50% tissue culture infective dose (TCID_50_) assay. Briefly, 96-well plates were seeded with 2 × 10^4^ MDCK cells/well the day before inoculation. The cells were washed with sterile PBS, followed by the addition of 100 μl of gradient dilutions of virus in maintenance medium (Opti-MEM [Invitrogen] with 0.3% BSA and 1 μg/ml TPCK-treated trypsin). The assay was carried out in eight parallels, with the last column of a 96-well plate as a control without virus. The viral titers were determined and calculated according to the Reed-Muench method.

### Coimmunoprecipitation and immunoblotting.

Cells were collected in lysis buffer (50 mM Tris, pH 7.5, 150 mM NaCl, 1 mM EDTA, and 0.05% NP-40 supplemented with PIC) and incubated with anti-Flag or anti-Omni antibody, together with protein A/G agarose (Sigma), at 4°C for 3 h. The beads were washed with ice-cold lysis buffer 5 times. The precipitates were mixed with 2× SDS sample buffer (100 mM Tris-Cl, pH 6.8, 20% glycerol, 4% SDS, 10% β-mercaptoethanol, and 0.12% bromophenol blue) and heated (96°C for 10 min), followed by Western blot analysis. For immunoblotting, the coimmunoprecipitation sample or 2% whole-cell lysates were analyzed by SDS-PAGE and transferred to a polyvinylidene difluoride (PVDF) membrane (Pall Corp.). The membrane was blocked in 3% skim milk in phosphate-buffered saline with Tween 20 (PBST) for 1 h and then incubated with primary antibody at 4°C overnight and anti-mouse or anti-rabbit IgG antibody conjugated to horseradish peroxidase (Millipore).

### GST pull-down assay.

GST-tagged PA, GST protein alone, and His-tagged HDAC6 were expressed in Escherichia coli BL21(DE3). GST fusion proteins were incubated with glutathione-coated beads for 2 h at 4°C, and His-tagged HDAC6 was purified on a nickel affinity chromatography column. The purified HDAC6 was mixed with GST-fused proteins bound to glutathione-coated beads in E1A binding buffer (50 mM HEPES, pH 7.6, 50 mM NaCl, 5 mM EDTA, 0.1% NP-40, and 10% glycerol) and incubated for 4 h at 4°C. After washing 5 times in E1A binding buffer, samples were treated with 2× SDS sample buffer (40).

### Knockdown of HDAC6 expression.

siRNA oligonucleotides against HDAC6 and a nontargeting control siRNA were purchased from Biotend. For siRNA gene knockdown experiments, cells were cultured in 6-well plates and transfected with 10 nM 5 μl lipofectamine 2000 (Invitrogen) according to the manufacturer’s instructions. After 48 h of transfection, the cells were analyzed to determine the knockdown efficiency. The HDAC6 RNA interference (RNAi) target sequences were as follows: no. 1, CACCGTCAACGTGGCATGGAA (41), and no. 2, GGTGTCACCTGAGGGTTATAA.

### Immunofluorescence microscopy.

MDCK cells were grown on coverslips and cotransfected with pEGFP-HDAC6 and pcDNA3-Flag-PA or transfected with pEGFP-HDAC6, followed by virus infection. The cells were fixed and permeabilized with 4% formaldehyde and 0.1% Triton X-100 at 37°C for 30 min. After washing with glycine-PBS, the cells were blocked with 3% BSA in PBS for 1 h at room temperature. The coverslips were incubated with primary antibody (1:200) for 1 h, followed by secondary antibody (1:500; Life Technologies) for 30 min. Unbound antibodies were washed thrice with PBST. The slides were stained with DAPI (4′,6-diamidino-2-phenylindole) containing the anti-fade Dabco solution (Sigma). Subcellular localization images were obtained with a confocal microscope (Nikon), and other images were obtained under a Nikon fluorescence microscope (TS100-F; DSRi2).

### RNA isolation, Q-RT-PCR, and RT-PCR.

Total RNA was isolated from cells with TRIzol reagent (Invitrogen). The cDNA was amplified with reverse transcriptase (Vazyme) according to the manufacturer’s guidelines. Specific primers were used for viral segment 5 cDNA synthesis, and then the NP vRNA and mRNA levels were quantitated. SYBR green-based quantitative real-time PCR was performed using a Life Technology instrument. The parameters for amplification were 95°C for 30 s and 40 cycles of 95°C for 10 s and 60°C for 30 s, followed by a melting curve analysis. The fold changes in mRNA and vRNA were calculated using the ΔΔ*C_T_* method. The primers used in the study are listed in Table 1.

**TABLE 1:**
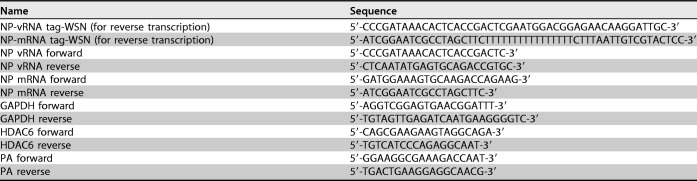
Primers used for reverse transcription, RT-PCR, and Q-RT-PCR

### Luciferase minigenome reporter assay.

The dual-luciferase assay (Promega, Madison, WI, USA) was performed in triplicate according to the manufacturer’s instructions. To examine how tubacin affects IAV RNA polymerase activity, MDCK cells were cultured in 24-well plates and cotransfected with an IAV minigenome reporter, pPolI-NP-firefly (200 ng), and an internal control *Renilla* luciferase assay vector, pCMV-RL (3 ng), along with plasmids expressing IAV RNA polymerase complex proteins (PA, PB1, PB2, and NP; 50 ng each) for 8 h and subsequently left untreated (dimethyl sulfoxide [DMSO]) or treated with tubacin (10 μM) for 20 h. To detect luciferase activity during IAV infection, MDCK cells were cotransfected with pPolI-NP-firefly (200 ng) and pCMV-RL (3 ng) for 24 h, followed by treatment with DMSO or tubacin for 2 h, and subsequently infected with WSN for 3 h. Luciferase activity was measured with a dual-luciferase kit and a Turner Designs luminometer. The fold change in relative luciferase activity was a product of the luciferase activity induced by tubacin divided by that induced by DMSO.

### Mass spectrometric analysis.

Samples were run in one-dimensional SDS-PAGE, and potential PA-containing bands were cut out and subjected to in-gel tryptic digestion overnight. Experiments were performed on a Q Exactive mass spectrometer that was coupled to an Easy nano-liquid chromatography (nLC) instrument (Thermo Fisher Scientific). The peptide mixture was loaded onto a C_18_ reverse-phase column (15 cm long; 75-μm inside diameter) packed in house with RP-C_18_ 5-μm resin in buffer A (0.1% formic acid in high-performance liquid chromatography [HPLC[ grade water) and separated for >60 min with a linear gradient of buffer B (0.1% formic acid in 84% acetonitrile) at a flow rate of 250 nl/min controlled by IntelliFlow technology. The MS data were acquired using a data-dependent top10 method (the first top 10 peptide peaks), dynamically choosing the most abundant precursor ions from the survey scan (300 to 1,800 *m/z*) for higher-energy collisional HCD fragmentation. Determination of the target value is based on predictive automatic gain control (pAGC). The dynamic-exclusion duration was 20 s. Survey scans were acquired at a resolution of 70,000 at *m/z* 200, and the resolution for the higher-energy collisional dissociation (HCD) spectra was set to 17,500 at *m/z* 200. The normalized collision energy was 27 eV, and the underfill ratio, which specifies the minimum percentage of the target value likely to be reached at maximum fill time, was defined as 0.1%. The instrument was run with peptide recognition mode enabled. Tandem mass spectrometry (MS-MS) spectra were searched using the MASCOT engine (Matrix Science, London, United Kingdom; version 2.2) against the target protein sequence. For protein identification, the following options were used: peptide mass tolerance, 20 ppm, MS-MS tolerance, 0.1 Da; enzyme, trypsin; missed cleavage, 2; fixed modification, carbamidomethyl (C); variable modification, oxidation (M); acetyl (K); ion score, >20.

### Statistical analysis.

The results shown are representative of experiments repeated a minimum of three times. Statistical comparisons were done with Student's *t* test. A *P* value of less than 0.05 was considered statistically significant.

## Supplementary Material

Supplemental file 1
