# Supplementary material for: HDAC6 Restricts Influenza A Virus by Deacetylation of the RNA Polymerase PA Subunit
Source: J Virol. 2019 Feb 5;93(4):e01896-18. doi: 10.1128/JVI.01896-18 (PMC6364008; doi:10.1128/JVI.01896-18)
Supplement: Supplemental file 1 [file b181ec2c923208acfb2e808b64551956_JVI.01896-18-s0001.pdf]

Figure S1

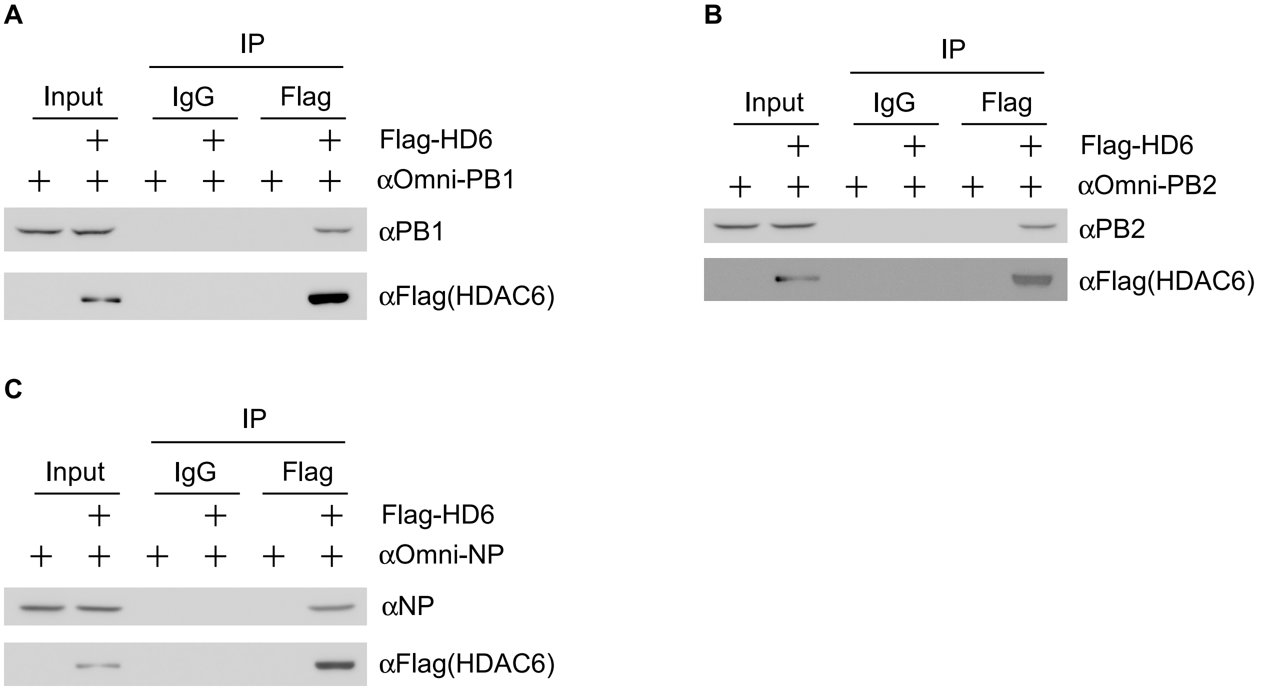

Fig.S1. HDAC6 interacts with PB1, PB2 and NP, subunits of the IAV RNA polymerase complex

(A) 293T cells were co-transfected with Flag-HDAC6 or empty vector and Omni-PB1, and whole cell lysates were immunoprecipitated by Flag antibody or control IgG. The immunocomplexes were then used to detect Omni-PB1 and Flag-HDAC6 by immunoblotting with the indicated antibodies. (B) The experiment was performed as in panel A, except Omni-PB2 were transfected. (C) The experiment was performed as in panel A, except Omni-NP was transfected.

Figure S2

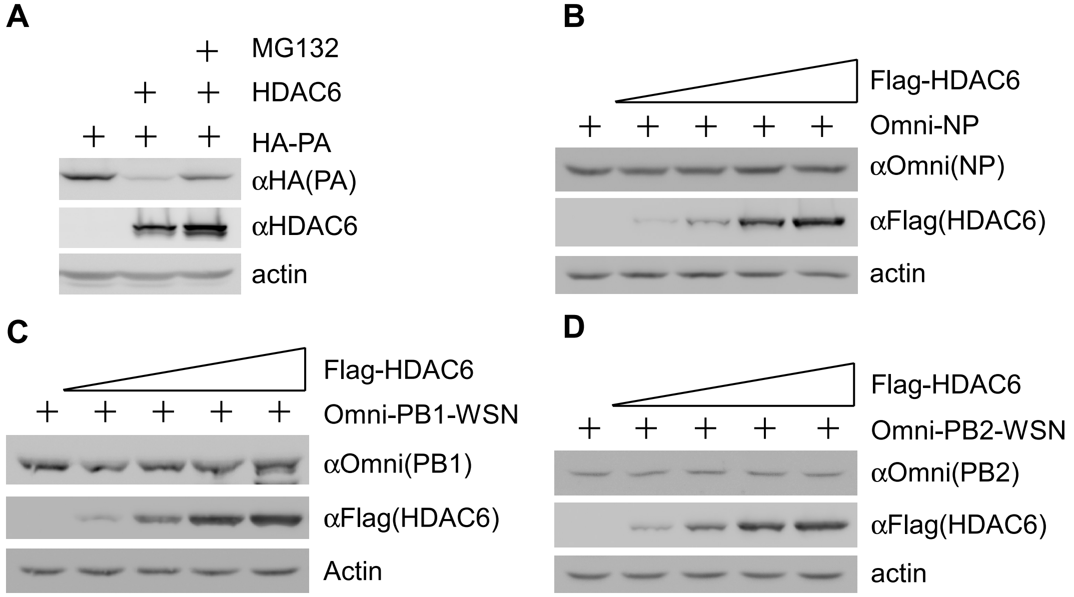

Fig.S2. HDAC6 has no effect on viral protein NP, PB1 and PB2 expression level

(A) HA-PA were co-transfected with control vector or HDAC6, and MG132 (10 μM) was treated 5 hours before collecting sample as indicated. PA, HDAC6 and actin were detected by immunoblotting. (B) 293T cells were co-transfected with a fixed amount (500 ng) of Omni tagged NP and a gradient of increasing amounts of Flag tagged HDAC6. The cell lysate were collected and detected by immunoblotting with the indicated antibody at 36 hours post transfection. (C) The experiment was carried out as in panel (B), except Omni tagged PB1 was transfected instead of NP. (D) The experiment was carried out as in panel (B), except Omni tagged PB2 was transfected instead of NP. (E) 293T cells were transfected with Flag-tagged PA. After immunoprecipitation of anti-Flag antibody, the immunocomplex was separated and visualized by Coomassie blue stain. The bands corresponding to PA and acetylated PA were cut out of the gel and then subject to mass spectrometry. The mass spectrometry acetylation modification peak map is shown. (F) The experiment was performed as shown in panel E except the ubiquitination modification was detected and analyzed.

Figure S3

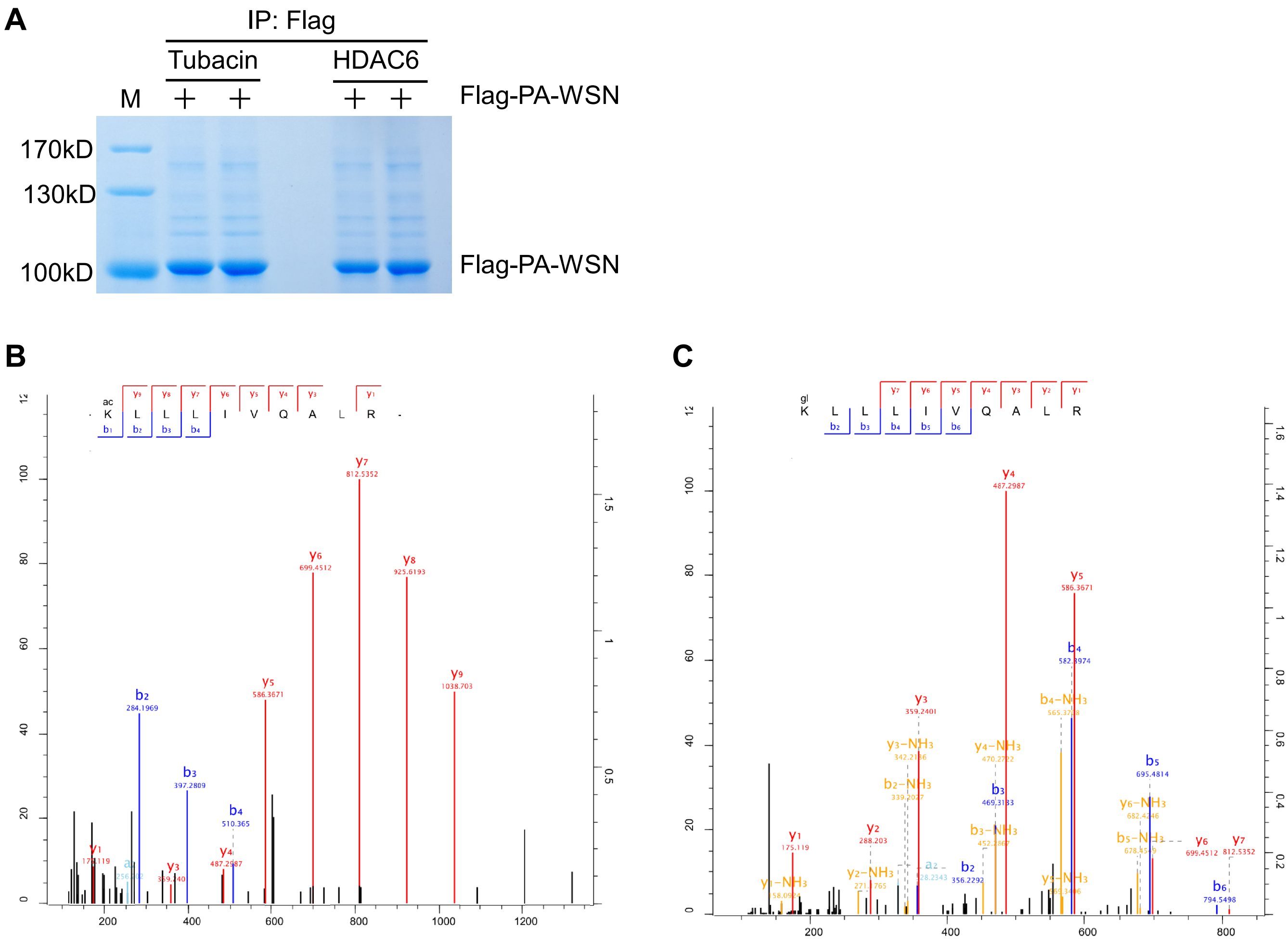

Fig.S3. Coomassie staining gel for mass spectrometry.

(A)293T cells were transfected with Flag-tagged PA or HDAC6 seperately. The cells were then collected for immunoprecipitation followed by 36 hours transfection, treated with Tubacin or co-incubated with HDAC6. After immunoprecipitation of anti-Flag antibody, the immunocomplex was separated and visualized by Coomassie blue stain. The bands corresponding to PA and acetylated PA were cut out of the gel and then subject to mass spectrometry. (B-C) Samples were subjected to in-gel tryptic digestion and analyzed by mass spectrometry as described in Materials and Methods. Representative peptide sequences of PA are shown at the top of the mass spectra. This analysis revealed that The Lys(664) residue of PA protein can be acetylated (B) and ubiquitinated (C).

Figure S4

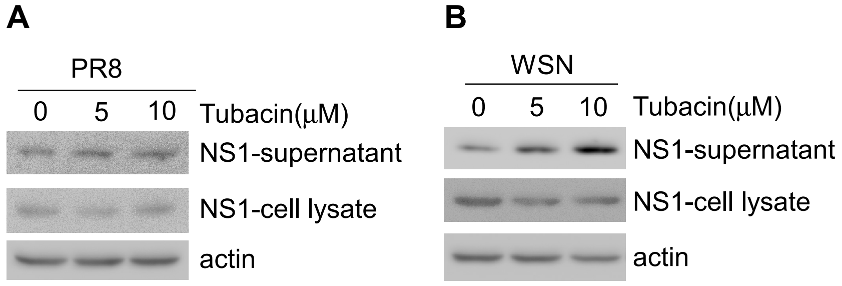

Fig.S4. Inhibition of HDAC6 activity promotes IAV (WSN and PR8) releasing stage

(A) MDCK cells were infected with PR8 (1 MOI) and treated with Tubacin. Twenty-four hours after infection, the culture medium and cell lysate were prepared for immunoblotting. NS1 in the culture medium and cells and actin in the cells were detected. (B) The experiment was performed as in panel A, except using the WSN virus.

Figure S5

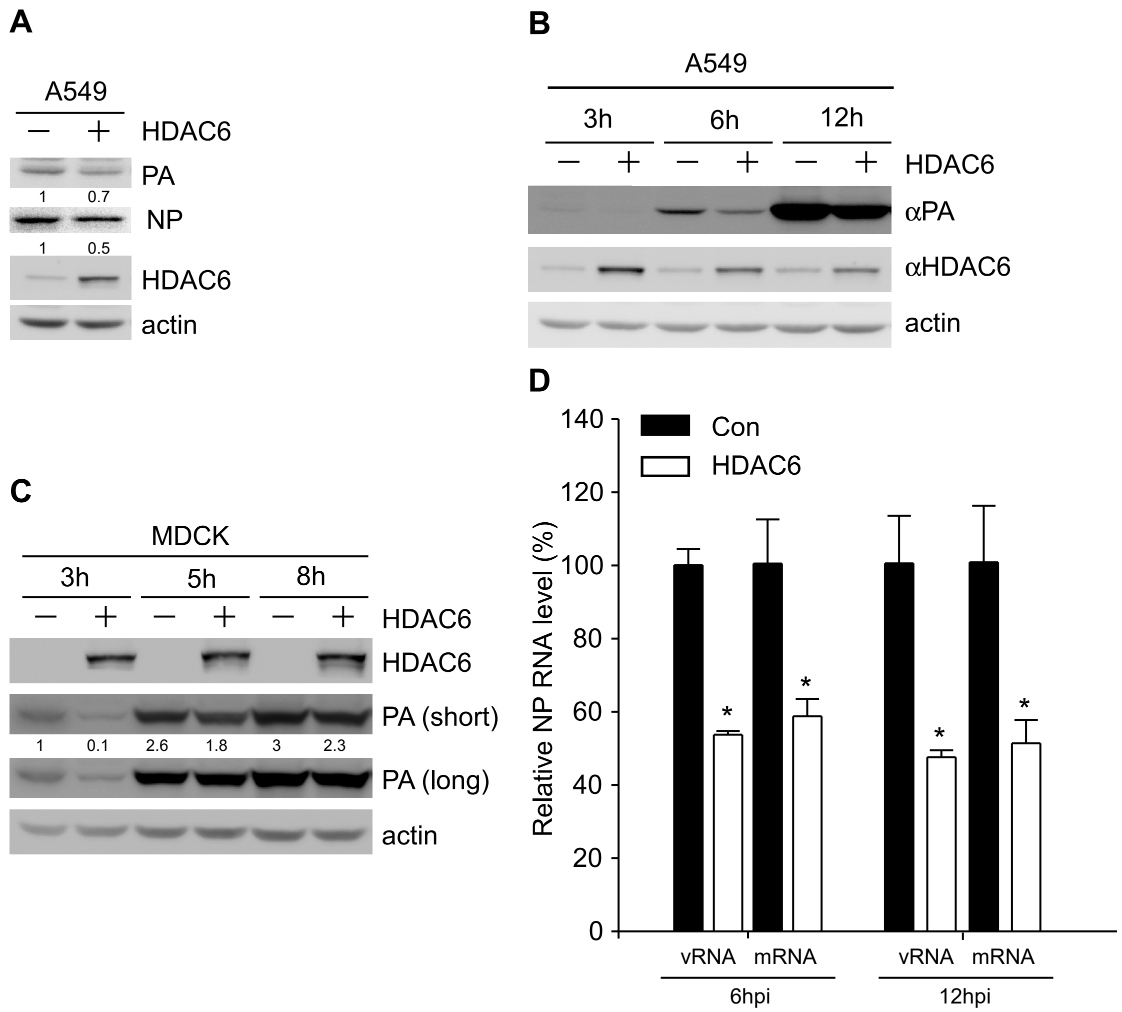

Fig.S5. HDAC6 inhibits IAV infection

(A) A549 cells were transfected with HDAC6 or empty vector for 36 hours followed by WSN infection (2 MOI) for 3 hours. PA, HDAC6 and actin were detected by immunoblotting. (B) A549 cells were transfected with HDAC6 or empty vector for 36 hours, followed by WSN infection at 1 MOI for the indicated time points. Cells were collected for immunoblotting. (C) The experiment was performed as in panel B, except MDCK cells were used and different virus infection time. (D) MDCK cells were transfected with HDAC6 or empty vector for 36 hours followed by WSN infection (2 MOI) for the indicated time points. NP mRNA and vRNA levels were quantified by Q-RT-PCR.

Figure S6

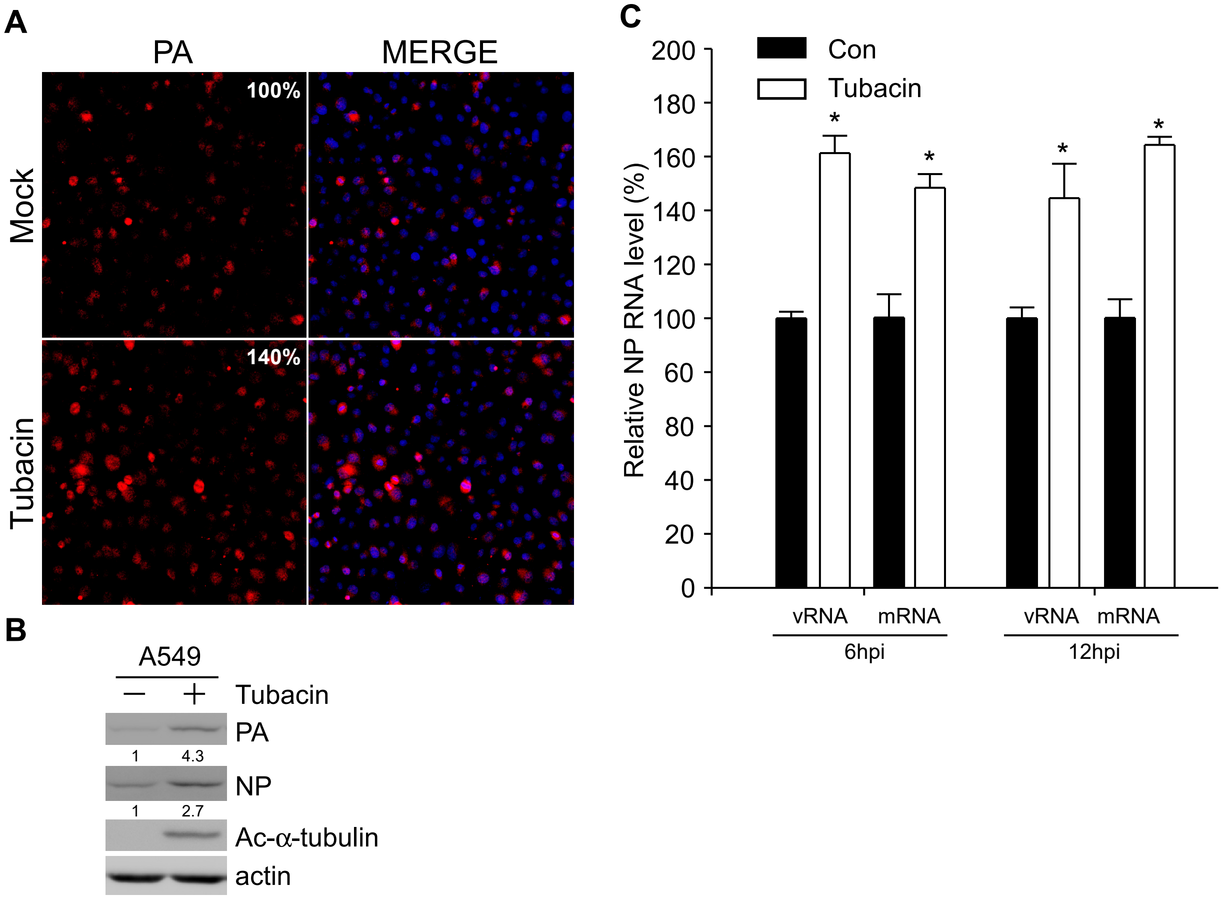

Fig.S6. Inhibition of HDAC6 promotes IAV infection

(A) MDCK cells were pre-treated with Tubacin (1 μM) for 2 hours and subsequently infected with WSN (2 MOI) for 3 hours. The cells were fixed and stained with PA antibody and Alexa 555-conjugated goat anti-rabbit IgG antibody (red). Nucleus was stained with DAPI (blue). The percentage of PA relative amount was calculated. (B) The experiment was performed as in panel A except A549 cells were used. Cell lysates were prepared for immunoblotting to detect PA, NP, ac-α-tubulin, α-tubulin and actin.(C) MDCK cells were pre-treated with Tubacin (1 μM) for 2 hours and subsequently infected with WSN (2 MOI) for the indicated time points. The levels of NP mRNA and vRNA were quantified by Q-RT-PCR.
